# Patient-centered AI

**DOI:** 10.3389/fdgth.2025.1638098

**Published:** 2025-08-13

**Authors:** Shamie Kumar

**Affiliations:** Department of Clinical Research, Qure.ai Technologies Private Limited, London, United Kingdom

**Keywords:** artificial intelligence (AI), patient-centered, patient anxiety, lung cancer, early diagnoses, ethical and regulatory, implementation challenges

Despite advances in diagnostics and treatment, healthcare systems worldwide remain largely reactive. Care is typically initiated only after the onset of symptoms, leading to avoidable delays and worse patient outcomes. This issue is especially pronounced in oncology, where late-stage diagnoses are common and often fatal. Lung cancer remains a significant public health concern (1), with delayed diagnosis being a major contributor to low survival rates (2, 3). In early-stage lung cancer, a treatment delay of just 1 week is associated with an increased mortality risk ranging from 1.2% to 3.2%(4).

A meaningful shift occurs when technology is designed around patient needs rather than institutional workflows. In this article, we explore how artificial intelligence (AI), when thoughtfully deployed, can support earlier diagnoses and enhance patient-centered care using qXR as an example.

AI solutions such as qXR from Qure.ai have been integrated into routine chest x-rays (CXRs) to enhance the early detection of lung cancer, including asymptomatic and subtle cases, improving diagnostic accuracy and supporting early diagnosis. In a multicenter study conducted across eight sites, AI identified missed findings in 23.3% of CXRs that were initially reported as normal by radiologists. When used as a second reader, AI increased the overall sensitivity from 64.7% to 75.4%, with only a modest reduction in specificity, from 98.3% to 95.2%. Reader confidence improved in more than two-thirds (68.3%) of cases involving missed abnormalities, underscoring the role of AI in improving both diagnostic accuracy and interpretive certainty (5). A separate large-scale retrospective study involving CXRs from 40 hospitals evaluated AI's performance as a second reader for lung nodule detection among both radiologists and non-radiologist physicians. The results showed that AI assistance significantly improved sensitivity—by up to 23.6% for non-radiologists and 17.4% for radiologists. It also led to a 40% reduction in missed findings, a 30% improvement in localization accuracy, and shortened interpretation time by an average of 12–15 s per case (6).

In another validation study conducted across 45 locations, AI demonstrated superior diagnostic performance compared to radiologists. It achieved a sensitivity of 92.3% and a specificity of 85.1%, outperforming human readers, who recorded a sensitivity of 84.6% and a specificity of 77.4% (7).

These findings demonstrate that AI not only enhances diagnostic accuracy and reader confidence but also streamlines interpretation workflows by reducing missed findings and expediting time to diagnosis. When embedded into standard practice, AI equips clinicians to identify disease such as lung cancer earlier, enabling more timely interventions and improving patient outcomes.

We now turn to shift from reactive to proactive care, and what makes this truly patient-centered is not just the use of cutting-edge AI, but how it enhances the patient's pathway in a practical manner:

• *Reduced waiting times*: AI processes chest x-rays immediately following acquisition, detecting and triaging findings suspicious for lung cancer to enable faster reporting. In the RADICAL study (8), researchers evaluated the integration of qXR into NHS workflows and reviewed how AI-enhanced triage has reduced average reporting times and expedited CT follow-ups for suspected lung cancer cases, especially in resource-constrained hospitals.
• *Earlier detection*: Patients who might otherwise remain undiagnosed for months or years can be identified earlier, leading to better outcomes (2, 3, 5). AI-assisted CXRs have demonstrated the potential to detect lung cancer at earlier stages, including in patients who may have otherwise remained undiagnosed for extended periods, thereby improving prognosis and enabling timely intervention (9). This is particularly impactful in healthcare systems serving large, diverse populations, where diagnostic delays are more common due to staffing and capacity constraints.
• *Minimizing patient anxiety*: Faster, shorter, and clearer diagnostic pathways help reduce the mental burden often associated with uncertainty and delays. A meta-analysis by Wang et al. (10) showed a significant correlation between diagnostic delays and psychological distress in cancer patients. Similarly, Nixon et al. (11) found that shorter diagnostic intervals were associated with improved psychological outcomes across multiple cancer types.

## Principles of patient-centered AI

The promise of AI is not without risks. Poorly designed algorithms can perpetuate health inequities, and over-reliance on these systems may undermine clinical accountability. Several key principles of patient-centered AI are beginning to emerge:

• *Transparency and trust*: Patients should be informed when AI is involved in their care and understand how it contributes to decision-making. Clinicians must remain the ultimate decision-makers, ensuring that AI serves as an augmentation, not a replacement (12–14).
• *Equity*: A patient-centered approach ensures that the benefits of AI are distributed equally across all demographic groups, without favoring any certain population. Broader deployment should aim to detect cancers irrespective of socioeconomic background (13).
• *Holistic support*: Patient-centered AI goes beyond diagnosis, supporting the emotional, logistical, and social aspects of the patient journey. By enabling quicker diagnoses and more streamlined care pathways, it supports faster interventions, improves mental health outcomes, and strengthens the patient–clinician relationship (9, 15).

## Challenges and implementation barriers

While AI holds immense promise, its real-world implementation faces several challenges, with cost and sustainability being persistent concerns. Although AI can help reduce downstream costs by enabling earlier diagnosis, the initial outlay, including licensing, infrastructure, and training, can be prohibitive, particularly for public or non-profit healthcare providers (16) and for under-resourced or rural regions. Successful deployment of AI tools often depends on stable internet connectivity, robust IT imaging infrastructure, trained personnel, and streamlined referral pathways. However, in many low- and middle-income countries, these foundational systems are fragmented, inconsistently funded, or entirely absent (2, 3, 12).

Even in high-income healthcare systems, clinician trust and acceptance remain critical. Zhou et al. (15) highlighted that explainability is a key factor influencing whether clinicians choose to rely on AI tools. A lack of transparency can erode confidence and lead to reluctance in their adoption. In addition, disparities in AI performance across ethnic or demographic groups raise concerns about fairness, especially when models are trained on non-representative datasets (17). Workflow integration is another hurdle. AI tools that require separate platforms or contradict clinical intuition can disrupt care and increase workloads (18). Integration with existing electronic health records (EHRs) and imaging workflows is important to maximize usability and clinical benefit.

The use of AI in clinical care is advancing rapidly, but the legal, regulatory, and ethical frameworks governing its deployment remain in flux. In many jurisdictions, questions of accountability, particularly in cases of diagnostic errors arising from AI decision-making, are still not clearly defined (13). At the same time, data privacy regulations, while essential for protecting patient confidentiality, may inadvertently restrict the data sharing necessary to enhance algorithmic performance and generalizability (14). Despite ongoing challenges, the clinical value of AI across diverse healthcare settings remains evident. Autonomous AI applications are gaining acceptance for well-defined use cases. Notably, the World Health Organization has endorsed computer-aided detection (CAD) software as a viable autonomous alternative to human interpretation of chest radiographs in resource-limited settings, particularly for the detection of tuberculosis, where radiological expertise is often scarce (2, 3).

## Patient-centered deployment

Effective deployment of AI in clinical care must extend beyond technical accuracy to deliver tangible improvements in patient experience and health outcomes. A patient-centered approach ensures that the benefits of AI are translated into care that is timely, equitable, and person-focused (15).

This shift involves a proactive reconfiguration of the diagnostic and treatment pathway, including

• *Earlier intervention opportunities*: AI-driven detection of subtle abnormalities on CXRs enables faster patient recalls, streamlined referrals, and optimized diagnostic scheduling (6, 7).
• *Improved access to downstream diagnostics and care*: This includes expedited same-day or next-day CT imaging, rapid “hot” reporting, and earlier approach into multidisciplinary team (MDT) reviews—particularly critical in high-volume or underserved systems (8, 18).

By enabling earlier diagnosis and reducing unnecessary delays, these AI-driven enhancements can help patients avoid the invasive and costly treatments associated with late-stage disease (4); at the same time, they promote a care experiences that feels less reactive and more collaborative, transforming the patient journey into a true healthcare partnership rather than a crisis-driven response (2, 3, 5).

## Integrating AI into patient journeys: A blueprint for the future

• *Seamless integration into primary and community care settings*: Expanding AI deployment beyond tertiary hospitals is essential, particularly in resource constrained or high-throughput environments where diagnostic delays are more common and early detection can significantly improve outcomes (2, 3, 5). AI tools have demonstrated value in supporting earlier identification of lung cancer in asymptomatic individuals, even in routine care settings, thereby contributing to improved prognosis and survival (6, 9).
• *Ongoing education for clinicians and patients*: Continuous education is critical to ensure safe, appropriate, and confident use of AI technologies. This includes raising awareness of AI's capabilities and limitations, as well as addressing challenges related to its interpretability (14, 17).
• *Embedding feedback loops*: Incorporating real-world input from both clinicians and patients informs into the iterative development of AI tools can ensure that such systems remain clinically useful, ethically sound, and socially acceptable (15).
• *Cross-sector collaboration*: Such collaboration must underpin AI integration. Partnerships among technologists, clinicians, regulators, researchers, and patient representatives are essential to ensure that innovation remains aligned with patient-centered values and adheres to broader ethical and legal responsibilities (12, 13, 18).

These elements ensure that AI not only improves outcomes but also fosters trust, compassion, and continuity in care.

## Conclusion

Patient-centered AI creates a system where technology amplifies, rather than replaces, human care. By enabling earlier diagnoses, faster pathways, and more personalized care, it offers patients not only better outcomes but better lives. When AI is deployed thoughtfully, ethically, and with the patient at the center of every decision, we move closer to a model of care where innovation serves people first.
